# Cerebellar hemorrhage in neonates: pattern analysis by ultrasonography and magnetic resonance imaging

**DOI:** 10.1007/s00247-024-06126-w

**Published:** 2025-01-04

**Authors:** Gayoung Choi, Young Hun Choi, Seul Bi Lee, Yeon Jin Cho, Seunghyun Lee, Jung-Eun Cheon, Seung Han Shin, Bo-Kyung Je

**Affiliations:** 1 Department of Radiology, Korea University Ansan Hospital, Korea University College of Medicine, Ansan, Republic of Korea; 2 Department of Radiology, Seoul National University Children’s Hospital, Seoul National University College of Medicine, 101, Daehak-Ro, Jongno-Gu, Seoul, 03080 Republic of Korea; 3 Institute of Radiation Medicine, Seoul National University Medical Research Center, Seoul, Republic of Korea; 4 Department of Pediatrics, Seoul National University Children’s Hospital, Seoul National University College of Medicine, Seoul, Republic of Korea; 5 Innovative Medical Technology Research Institute, Seoul National University Hospital, Seoul, Republic of Korea

**Keywords:** Cerebellar hemorrhage, Cerebellum, Infant, Newborn, Magnetic resonance imaging, Ultrasonography

## Abstract

**Background:**

Cerebellar hemorrhage in neonates is increasingly being identified but is still underdiagnosed. While magnetic resonance imaging (MRI) is the optimal imaging modality for cerebellar hemorrhage evaluation, ultrasonography (US) is commonly used for screening. Characterizing the patterns and distribution of cerebellar hemorrhage lesions can help facilitate its detection by aiding to focus on prevailing type of cerebellar hemorrhage.

**Objective:**

This study aimed to analyze the patterns of cerebellar hemorrhage in neonates, comparing US findings with MRI.

**Materials and methods:**

This was a retrospective study of 765 neonatal intensive care unit (NICU)-admitted neonates who underwent brain MRI due to various clinical and radiological requirements. Two pediatric radiologists reviewed brain MRI and US in consensus, and cerebellar hemorrhage patterns were classified based on MRI findings: type 1, punctate cerebellar hemorrhage without cerebellar volume loss; type 2, focal cerebellar hemorrhage with cerebellar volume loss; type 3, ovoid/crescent cerebellar hemorrhage in the periphery of the cerebellar hemisphere; type 4, isolated vermian cerebellar hemorrhage; type 5, cerebellar hemorrhage involving almost the entire cerebellar hemisphere. The distribution and US detection rates of cerebellar hemorrhage were compared according to the cerebellar hemorrhage type.

**Results:**

A total of 56 (33 male, 23 female) cases (7.32%) among 765 MRIs showed cerebellar hemorrhage (median gestational age, 27 + 1 weeks [IQR 5 + 2]; median birth weight, 955 g [IQR 882.5]). The most common pattern was type 1 (60.7%). Type 3 cerebellar hemorrhage was more commonly observed in the inferior and peripheral cerebellum compared to types 1 and 2 cerebellar hemorrhage (*P*=0.002). In retrospective review of images, type 3 was the most commonly missed type of cerebellar hemorrhage (initial US detection rate, 33.3%; retrospective US detection rate, 75%).

**Conclusion:**

This study underscores the importance of understanding cerebellar hemorrhage patterns and suggests that careful inspection of inferior and periphery of the cerebellum is important to avoid missed diagnosis of cerebellar hemorrhage.

**Graphical Abstract:**

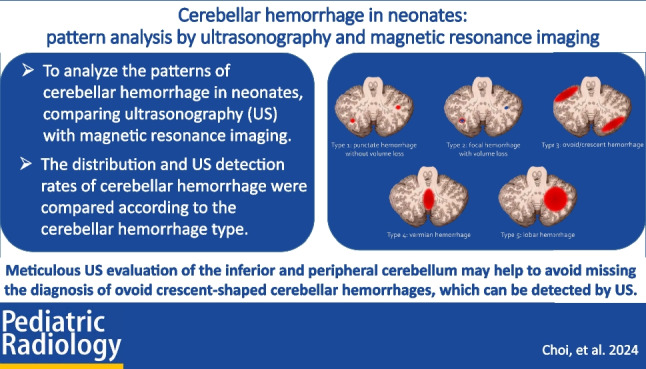

## Introduction

Cerebellar hemorrhage in neonates is not uncommon with a reported incidence of 10–25% in preterm neonates, and extremely low birth weight, patent ductus arteriosus, and emergency cesarean section delivery are known risk factors [1–5]. The developing cerebellum is more vulnerable to injury at the gestational age (GA) of 23–27 weeks as there is a rapid increase in the cerebellar volume during this period [6]. Hemorrhagic injury in immature cerebellum is increasingly being detected due to increased survival of very preterm infants and advances in neonatal brain imaging. Various neurodevelopmental outcomes of cerebellar hemorrhages including impairments in motor and balance coordination, as well as cognitive, learning, and behavioral functions, are known to be affected by their location and extent [2, 7–11]. However, cerebellar hemorrhage is still underdiagnosed and its impact on neurodevelopmental complications is often underestimated [7]. This underscores the significance of early and accurate diagnosis for effective management of neonatal cerebellar hemorrhage.

The highly vascularized and bleeding-prone germinal matrix gradually involutes from 24 to 30 weeks of gestation and lastly remains around the frontal horns, resulting in germinal matrix hemorrhage most commonly at the caudothalamic grooves of premature neonates [12]. The cerebellar external granular layer is also a germinal zone; therefore, cerebellar hemorrhage is also commonly found in this area [7, 13, 14].

Although magnetic resonance imaging (MRI) has superior sensitivity and provides additional information for evaluating cerebellar hemorrhage in neonates, ultrasonography (US) is recommended as the initial imaging modality for screening cerebellar hemorrhage in neonates [15–17]. However, in US evaluation, the infratentorial brain is less clearly visible through the anterior fontanelle compared to the supratentorial brain. The use of additional acoustic windows, such as mastoid and posterior fontanelles, can improve the sensitivity of US for detecting cerebellar hemorrhage in preterm neonates [2]. In addition, due to the operator-dependent nature of US, being more aware of the common patterns and sites typically affected by cerebellar hemorrhage can lead to a more focused examination and better detection rates.

Several studies have evaluated the radiologic findings of cerebellar hemorrhage in neonates [5, 6, 10, 11, 13, 18–20]. However, no study has performed an in-depth evaluation of the US findings of cerebellar hemorrhage in neonates and compared these with MRI findings. Therefore, the purpose of this study was to investigate the patterns and distribution of cerebellar hemorrhage in neonates and to evaluate the US findings and detection rate of cerebellar hemorrhage, using MRI as the reference standard.

## Materials and methods

### Subjects

The institutional review board of our hospital approved this retrospective study and the need for informed consent was waived off.

From January 1, 2010, to February 28, 2019, infants admitted to the neonatal intensive care unit (NICU) who underwent brain MRI were eligible for inclusion in this study. The specific indications for neonatal brain MRI in NICU patients were as follows: GA of 28 weeks or less; birthweight of 1500 g or less; any abnormalities detected in screening brain US; birth asphyxia or any other events during birth; as per clinicians’ requirements. After the exclusion of 12 infants with congenital anomalies or metabolic disorders, a total of 765 patients were enrolled (584 preterm neonates and 181 term neonates).

### Image acquisition

At our institution, all preterm infants with a birth weight of <1,500 g or a GA of <28 weeks underwent brain MRI either at the term or before discharge. In addition, brain MRI was performed earlier at the clinician’s discretion if clinically necessary or if significant abnormalities were found on the cranial US. The enrolled NICU infants underwent routine brain MRIs around term equivalent age. The MRI machine and routine protocols used in our institution were as follows: Avanto (Siemens Healthcare, Erlangen, Germany); axial T1-weighted spin-echo sequence; T2-weighted turbo spine-echo sequence; 3D magnetization-prepared rapid gradient-echo (MP–RAGE) sagittal sequence with axial and coronal reformation; axial diffusion-weighted images with *b* values of 0 and 1,000; apparent diffusion coefficient map; axial susceptibility-weighted imaging. To prevent movement during imaging, the neonates were sedated using oral chloral hydrate (25 mg/kg).

All enrolled patients underwent at least one cranial US (Logiq E9, GE Healthcare, Waukesha, WI; Philips iU22, Philips Medical Systems, Bothell, WA; Aixplorer, SuperSonic Imagine SA, Aix-en-Provence, France) during their stay in the NICU. The first cranial US was performed within 3 days of delivery, and serial follow-up US studies were performed every 1 or 2 weeks until discharge, at the discretion of the clinicians. All cranial US examinations were performed by certified pediatric radiologists. Coronal and sagittal images of the brain through the anterior fontanelle were routinely obtained using a small convex transducer (3–10 MHz) and a linear transducer (6–15 MHz). Additionally, transverse images of the posterior fossa were obtained through the mastoid fontanelle.

All analyzed data, along with representative images, supporting the findings of this study can be found in the paper and its supplementary materials.

### Image evaluation

Brain MRIs and all cranial US examinations were retrospectively reviewed in consensus by two certified pediatric radiologists (G.C. and Y.H.C. with 5 and 16 years of experience in pediatric radiology, respectively). Both radiologists were blinded to clinical information.

**Fig. 1:**
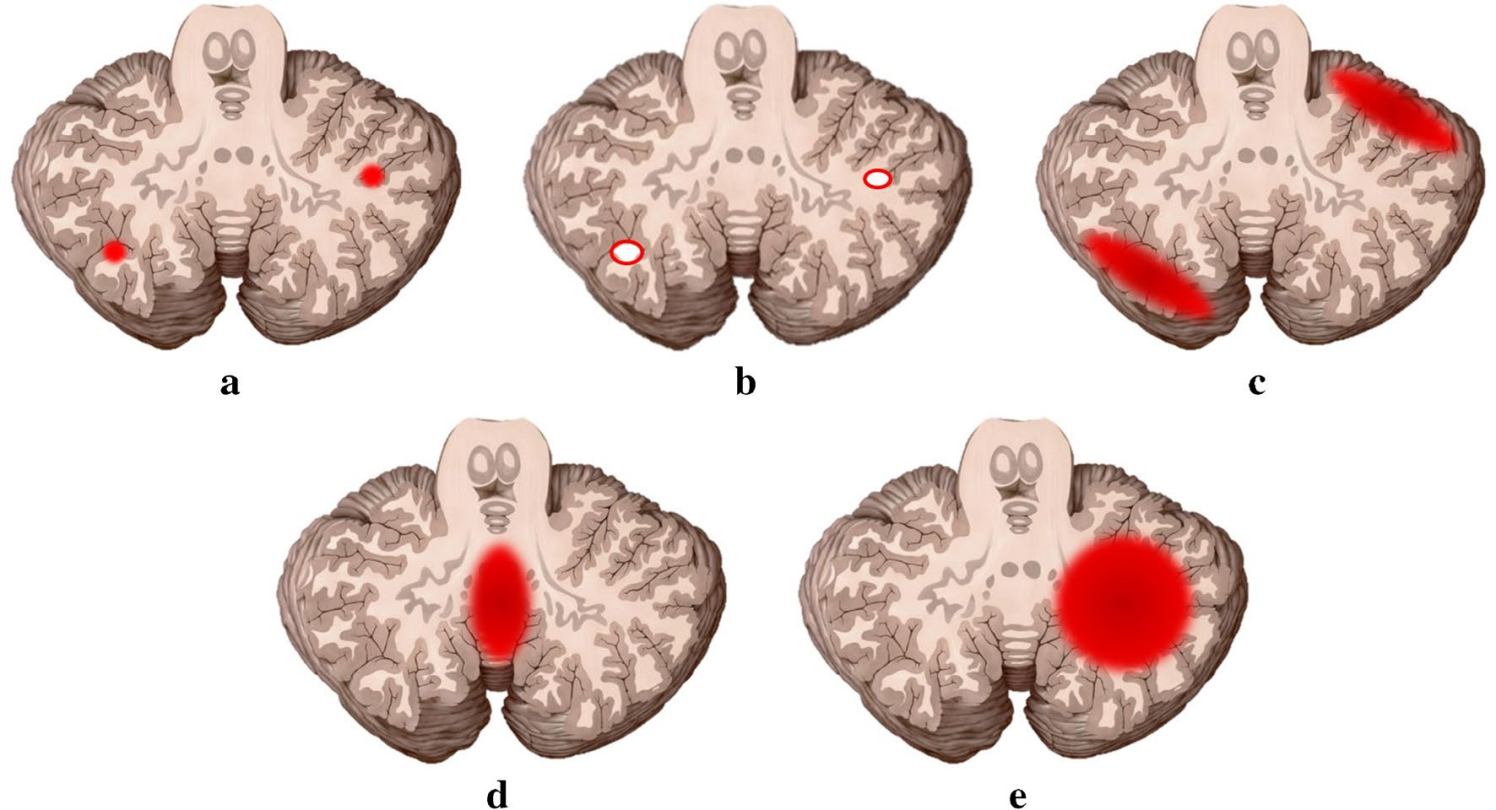
Schematic pictograms of cerebellar hemorrhage types. **a** Type 1, punctate hemorrhage without volume loss. **b** Type 2, focal hemorrhage with volume loss. **c** Type 3, hemispheric ovoid/crescent hemorrhage. **d** Type 4, vermian hemorrhage. **e** Type 5, large lobar hemorrhage

The pattern of cerebellar hemorrhage was classified into five types according to the location and extent of the cerebellar hemorrhage on MRI: type 1, punctate cerebellar hemorrhage without volume loss; type 2, focal cerebellar hemorrhage with volume loss; type 3, sizable ovoid/crescent cerebellar hemorrhage; type 4, hemorrhage in the cerebellar vermis; type 5, cerebellar hemorrhage involving almost the entire cerebellar hemisphere (Fig. 1). The locations of cerebellar hemorrhage were classified as follows: central (cerebellar white matter and deep nuclei) or peripheral (cerebellar cortex) or global; superior (above the primary fissure), mid (below the primary fissure and above the prepyramidal fissure), or inferior (below the prepyramidal fissure) or global (if two or more levels were involved). The supratentorial abnormalities were also evaluated.

On review of brain US images, cerebellar hemorrhage usually appeared as hyperechoic areas within the cerebellum. First, we calculated the detection rate of cerebellar hemorrhage according to the type of cerebellar hemorrhage, based on the formal radiology reports of the cranial ultrasonography. We then retrospectively reviewed the US images, reassessed the presence of cerebellar hemorrhage, and recalculated the detection rate.

### Statistical analysis

The incidences and types of the cerebellar hemorrhage were evaluated on brain MRI. The location predilection of cerebellar hemorrhage according to the type of cerebellar hemorrhage was analyzed using Fisher’s exact test. Correlations between the types of cerebellar hemorrhage and birth weight and GA were evaluated using the Spearman correlation coefficient with 95% confidence interval. Statistical analysis was performed using SPSS ver. 21.0 (SPSS Inc., Chicago, IL).

## Results

Among a total of 765 NICU-admitted neonates with brain MRIs, cerebellar hemorrhage was identified in 56 (7.32%) cases (male 33, female 23; median GA at birth 27 + 1 weeks [5 + 2]; median birth weight 955 g [882.5]). Thirty-two neonates (57.1%) had extremely low birth weight (<1,000 g), 9 (16.1%) had very low birth weight (<1,500 g), and 7 (12.5%) had low birth weight (<2,500 g). Twelve neonates were born at a GA of <25 weeks, 26 neonates were born between 25 and 28 weeks, 6 neonates were born between 28 and 32 weeks, 7 neonates were born between 32 and 37 weeks, and 5 neonates were born at ≥ 37 weeks.

Among 56 neonates with cerebellar hemorrhage, 23 neonates (41.1%) had isolated cerebellar hemorrhage, 33 neonates (58.9%) had concurrent supratentorial hemorrhage, 25 neonates (44.6%) had intraventricular hemorrhage (IVH), and 10 neonates (17.9%) had periventricular leukomalacia (PVL).

In pattern analysis of 56 cases of MRI-detected cerebellar hemorrhage, 34 cases (60.7%) were type 1, 4 (7.1%) were type 2, 12 (21.4%) were type 3, 2 (3.6%) were type 4, and 4 (7.1%) were type 5. The locations of the cerebellar hemorrhage according to the types are summarized in Table 1. In type 1 cerebellar hemorrhage, 24 (70.6%) cases involved the peripheral portion of the cerebellum and 14 (41.2%) cases occurred at the inferior level of the cerebellum. In type 3 cerebellar hemorrhage, all 12 cases occurred in the peripheral portion and 10 (83.3%) were inferior. Compared to type 1 cerebellar hemorrhage, type 3 cerebellar hemorrhage occurred significantly more frequently at the inferior level of the cerebellum (*P*=0.018) and the periphery (*P*=0.044). The inferior and peripheral location was involved in 29.4%, 50%, and 83.3% of cases with type 1, type 2, and type 3 cerebellar hemorrhages, respectively. When compared to type 1 and 2 cerebellar hemorrhage together, type 3 cerebellar hemorrhage more frequently occurred in the inferior and peripheral portions of the cerebellum (*P*=0.002).

**Table 1:** Distribution and location of cerebellar hemorrhage disaggregated by type

		Cerebellar hemorrhage type					Total
		1 (*n*=34)	2 (*n*=4)	3 (*n*=12)	4 (*n*=2)	5 (*n*=4)	
Bilaterality	Left	9	1	3	-	1	14
	Right	5	2	4	-	1	12
	Bilateral	20	1	5	-	2	28
Hemisphere/vermis	Hemisphere and Vermis	1	0	0	0	1	2
	Hemisphere only	33	4	12	0	3	52
	Vermis only	0	0	0	2	0	2
Location	Central	6	1	0	1	0	8
	Peripheral	24	2	12	0	0	38
	Global	4	1	0	1	4	10
Level	Inferior	14	2	10	1	0	27
	Mid	5	1	1	0	0	7
	Superior	11	0	1	0	0	12
	Global	4	1	0	1	4	10

There was inverse correlation between the type of cerebellar hemorrhage and birth weight (correlation coefficient, − 0.326; *P*=0.014) and GA at birth (correlation coefficient, − 0.320; *P*=0.016).

According to the formal US reports, 8 cases of cerebellar hemorrhage were found among 56 cerebellar hemorrhage cases (US detection rate 14.3%). In the retrospective review of US images, a total of 18 cerebellar hemorrhages were found (US detection rate 32.1%) (Table 2).

**Table 2:** Ultrasonography detection rates of cerebellar hemorrhage disaggregated by types

	Type 1	Type 2	Type 3	Type 4	Type 5
Case number (total 56)	34	4	12	2	4
Report-based detection rate (%)	0	25	33.3	50	50
Retrospective review-based detection rate (%)	2.9	50	75	100	100

**Fig. 2:**
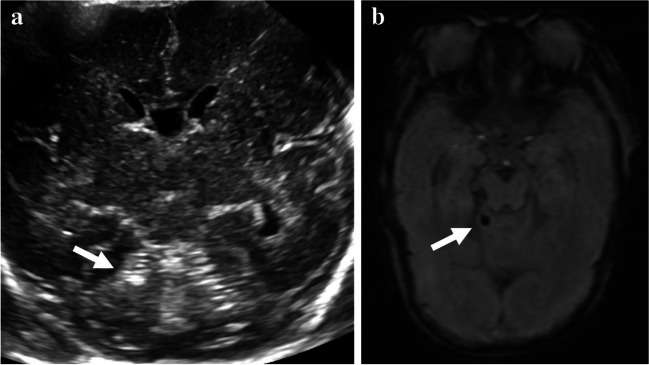
Initially missed but retrospectively detected type 1 cerebellar hemorrhage in a preterm boy (27 + 5 weeks, 1,080 g). **a** Oblique coronal anterior fontanelle view of cranial ultrasound image shows an echogenic punctate hemorrhage (*arrow*) in the right upper peripheral cerebellum, which was initially overlooked in the report, but was later discovered upon retrospective image review. **b** Axial susceptibility-weighted magnetic resonance image shows a tiny hemorrhagic focus (*arrow*) in the right upper peripheral cerebellar hemisphere

**Fig. 3:**
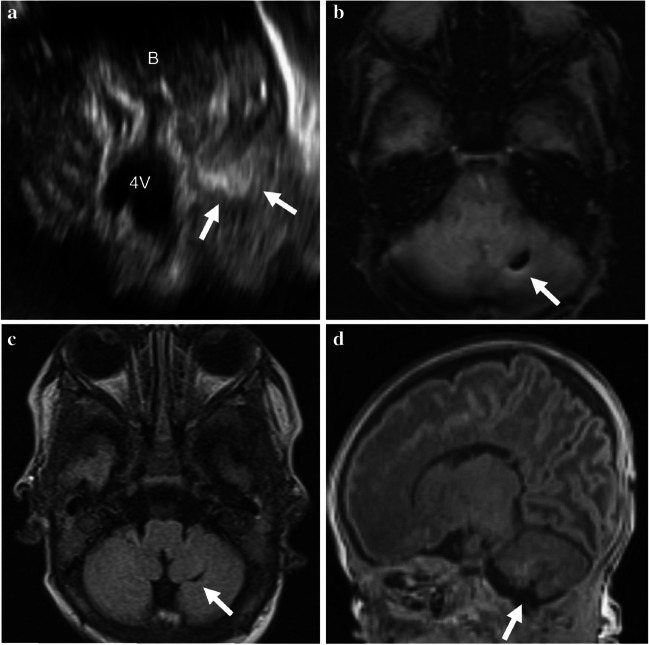
Representative case of type 2 cerebellar hemorrhage in a preterm girl (27 + 4 weeks, 980 g). **a** Axial right mastoid view of cranial ultrasound image (rotated 90° anticlockwise to align with the axial magnetic resonance image) shows an echogenic lesion (*arrows*) in the left inferior cerebellar cortex. **b** Axial susceptibility-weighted magnetic resonance image shows a corresponding dark signal lesion (*arrow*). **c**, **d** Unenhanced axial (**c**) and sagittal (**d**) T1-weighted magnetic resonance images show focal volume loss in the left inferior peripheral cerebellar cortex (*arrows*). B brain stem, 4 V fourth ventricle

**Fig. 4:**
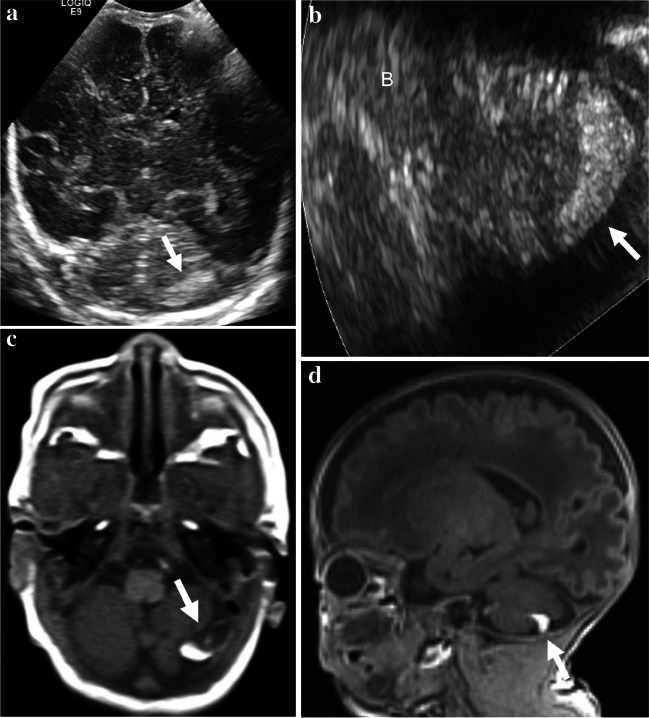
Representative case of type 3 cerebellar hemorrhage in a preterm boy (27 + 6 weeks, 880 g). **a**, **b** Oblique coronal anterior fontanelle view (**a**) and axial left mastoid view (**b**, rotated 90° clockwise to orient with the axial magnetic resonance image) of cranial ultrasound images show ovoid-to-crescent echogenic lesion (*arrows*) at the inferior periphery of the left cerebellum. **c**, **d** Unenhanced axial (**c**) and sagittal (**d**) T1-weighted magnetic resonance images obtained 7 weeks after the ultrasonography show T1-hyperintense hemorrhage (*arrow* in **d**) with focal cystic change (*arrow* in **c**)

**Fig. 5:**
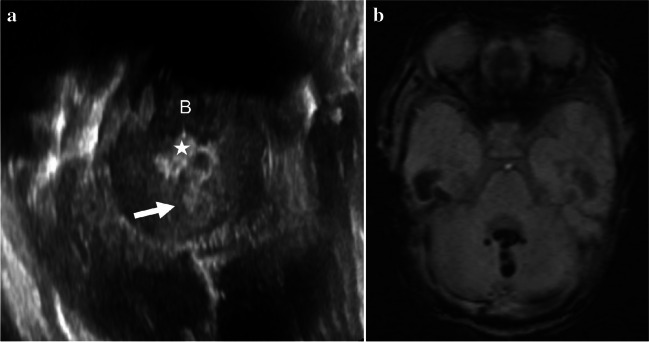
Representative case of type 4 cerebellar hemorrhage in a preterm boy (26 + 4 weeks, 530 g). **a** Axial left mastoid view of cranial ultrasound (rotated 90° clockwise to correspond with the axial MRI) shows cerebellar vermian hemorrhage (*arrow*) and echogenic intraventricular hemorrhage (*star*) in the fourth ventricle. **b** Axial susceptibility-weighted magnetic resonance image shows matching dark signal vermian hemorrhage and intraventricular hemorrhage. B brain stem

**Fig. 6:**
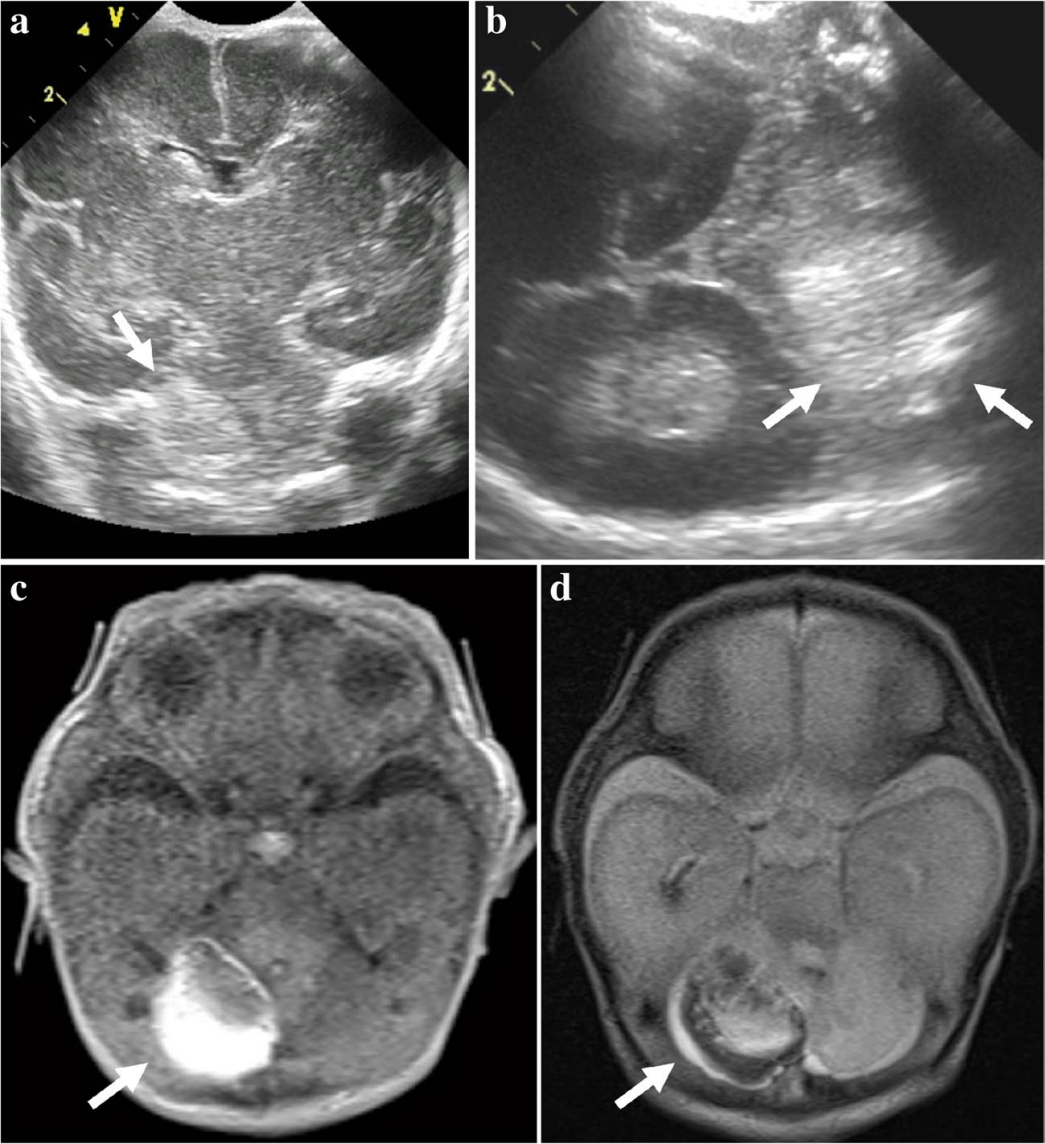
Representative case of type 5 cerebellar hemorrhage in a preterm girl (26 + 3 weeks, 1,020 g). **a**,** b** Oblique coronal anterior fontanelle (**a**) and axial left mastoid (**b**) views show large, mass-like echogenic lesion (*arrows*) in the right cerebellar hemisphere. **c** Axial T1-weighted magnetic resonance image acquired 10 days after the ultrasonography shows mixed high and iso-to-low signal intensity hemorrhage in the right cerebellar hemisphere. **d** Axial T2-weighted magnetic resonance image shows a complex lesion with mixed signal intensities in the right cerebellar hemisphere

The radiology report-based detection rates and retrospective review-based detection rates of cerebellar hemorrhages by types were as follows: 0% and 2.9%, respectively, in type 1; 25% and 50% (a twofold increase) in type 2; 33.3% and 75% (a 2.25-fold increase) in type 3; 50% and 100% (a twofold increase) in type 4; and 50% and 100% (a twofold increase) in type 5. Among the 34 cases of type 1 cerebellar hemorrhage (punctate hemorrhage without volume loss), none was detected in the US report and only 1 case was detected in the retrospective image review. In this initially missed but retrospectively detected case, punctate cerebellar hemorrhage appeared as a tiny echogenic lesion at the right upper peripheral cerebellar hemisphere (Fig. 2). Representative imaging findings of other different types of cerebellar hemorrhage are presented in Figs. 3, 4, 5 and 6.

## Discussion

Detection of cerebellar injury in neonates, particularly premature babies who are more vulnerable, is a key imperative because of its potentially adverse neurodevelopmental consequences [21]. Early and precise identification of cerebellar hemorrhage is helpful for diagnosis and treatment plans. The overall incidence of cerebellar hemorrhage in our study population was 7.32% according to the brain MRIs at term equivalent age, and the incidence showed an inverse correlation with birth weight and GA. Our study included preterm and full-term neonates admitted to the NICU. Therefore, the lower incidence of cerebellar hemorrhage, compared to prior studies that exclusively focused on preterm neonates [1–3, 13], may be attributed to differences in the target population.

In our study, cerebellar hemorrhage occurred more frequently in preterm infants and those with lower birth weight. Moreover, in more than half (58.9%) of the cases, the cerebellar hemorrhages were accompanied by supratentorial abnormalities. Such predilections have also been demonstrated in several previous reports [6, 9]. Therefore, it is important to keep in mind that extremely preterm infants, low birth weight infants, or infants with supratentorial abnormalities are at risk of developing cerebellar hemorrhage and should be carefully evaluated with cranial US.

Previous studies evaluating the impact of cerebellar hemorrhage on neurodevelopmental outcomes have used a broad categorization of cerebellar hemorrhage location as either unilateral or bilateral involvement of a hemisphere and/or the vermis, and have consistently reported that larger, bilateral lesions and broader distribution were associated with poorer outcomes [10, 19, 20, 22]. A summary of previous studies [5, 9, 11, 18, 19] and this study is listed in Table 3. However, such broad categorization is too simple and does not reflect the radiologic involvement patterns of cerebellar hemorrhage.

**Table 3:** The summary of previous studies of cerebellar hemorrhage in neonates and this study

Study	Study type	Imaging modality	Number of cerebellar hemorrhage	Total patient number	Categorization of cerebellar hemorrhage	Conclusion	Mean age	Mean birth weight
Limperopoulos et al., 2005 [5]	Prospective	MRI	10	74	Bilateral/unilateral	Cerebellar injury was associated with contralateral cerebral volume loss and deficits in cognition, behavior, and motor	26.7±2.3 weeks	972.2±250.7 g
Villamor-Martinez et al., 2019 [9]	Meta analysis	MRI, US	347	4,236	Focal/punctate	Neonates with cerebellar hemorrhage had lower GA and birth weight, and other comorbidities (hypotension, PDA, IVH, sepsis, NEC, BPD) Cerebellar hemorrhage was associated with delayed mental and psychomotor development, and increased incidence of cerebral palsy	NA	NA
Ecury-Goossen et al., 2010 [11]	Retrospective	US	15	3,201	Subarachnoid/folial/lobar/bilateral lobar/giant lobar including vermis/contusional	Motor agitation could be caused by cerebellar hemorrhage	25.3 weeks	730 g
Garfinkle et al., 2020 [18]	Prospective	MRI	36	234	Punctate/larger	Cerebellar hemorrhage at the inferior part of cerebellum showed the highest risk of adverse outcomes	27.7 weeks	NA
Boswinkel et al., 2019 [19]	Retrospective multicenter study	MRI	218	218	Punctate/limited/massive lateral-posterior-inferior/anteromedial/vermis	Larger cerebellar hemorrhage had more adverse outcome than limited cerebellar hemorrhage	27.2 weeks	958 g
Choi et al	Retrospective	MRI, US	56	765	Punctate hemorrhage without volume loss/focal hemorrhage with volume loss/hemispheric ovoid or crescent/large lobar	Careful US examination on inferior periphery of cerebellum is important to avoid missed diagnosis of potentially significant cerebellar hemorrhage	27.1 weeks	955 g

*BPD* bronchopulmonary dysplasia, *IVH* intraventricular hemorrhage, *MRI* magnetic resonance imaging, *NA* not applicable, *NEC* necrotizing enterocolitis, *PDA* patent ductus arteriosus, *US* ultrasonography

Compared to previous studies, which used broader and simpler categorizations of cerebellar hemorrhages, a more detailed classification that includes five distinct types to improve ultrasonography detection of cerebellar hemorrhage is suggested in this study

We classified the type of cerebellar hemorrhage into five categories based on the anatomical and pathological considerations, in addition to the MRI findings, reflecting the size and distribution of lesions. The main blood supply to the developing cerebellum is provided by the posterior inferior cerebellar artery, contributing to the higher prevalence of cerebellar hemorrhages in the inferior regions [18, 23] (especially types 3 and 4 in our classification). The external granular layer, the germinal matrix of the cerebellum, is located in the periphery of the cerebellum, making it prone to bleed in the peripheral cerebellum [13, 14] (as observed in types 1 and 3 in our classification). Furthermore, this classification reflects the size and extent of the hemorrhage, with smaller hemorrhages without volume loss are more likely to be missed but clinically insignificant, while larger lesions with a higher potential for sequelae are more readily detected.

Our new classification scheme of cerebellar hemorrhage showed an inverse correlation with birth weight and GA. In addition, the US detection rates showed a consistently increasing trend from type 1 through type 5 cerebellar hemorrhage. The detection rate of type 1 cerebellar hemorrhage was <5% while that of higher grades was ≥ 75%. We look forward to evaluating the clinical impact of our classification scheme in the future.

While the cranial US is commonly used for routine screening and follow-up of the neonatal brain, our results showed that its detection rate for cerebellar hemorrhage is far inferior to that of brain MRI. Particularly for small cerebellar hemorrhages, such as types 1 and 2 in our study, only 1 out of the 38 cases was initially detected, and 2 additional cases were detected in retrospective image review. Previous studies by Boswinkel et al. [19] and Brossard-Racine and Limperopoulos [10] have suggested that small cerebellar hemorrhages are clinically less significant. In our study, type 1 cerebellar hemorrhage, which was very challenging to detect by US, would have had little clinical impact based on the prior studies. On the contrary, type 3 cerebellar hemorrhage, the second most common type (21.4%) in our study, involves larger areas than types 1 and 2 and thus has greater clinical significance [10, 18, 19]. However, our study revealed that type 3 cerebellar hemorrhages were most frequently overlooked in the initial reports of the cranial US, with the most substantial improvement observed during retrospective review (only 4 cases were reported on the formal radiology reports, while 5 additional cases were identified during the retrospective review). This underscores the significance of prior knowledge regarding the US imaging features of cerebellar hemorrhages. In all cases of type 3 cerebellar hemorrhage, the lesion was located in the peripheral cerebellar hemisphere, predominantly in the inferior part. This positional predilection is in line with previous studies [18, 23, 24], and may be associated with the location of the germinal matrix of the cerebellum. The external granular layer, the germinal zone of the cerebellum located in the peripheral cerebellar hemisphere, is particularly vulnerable to hemorrhage. Notably, even higher vulnerability of the inferior and posterior area of cerebellar external granular layer is attributed to a combination of developmental and vascular factors: the anterior-to-posterior progression of external granular layer maturation, which results in delayed maturation of the posterior cerebellum; the late expression of *Math-1* in the posterior cerebellum, which prolongs its immature, proliferative state; and the relatively fragile blood supply from the posterior inferior cerebellar artery, where instability in blood flow regulation may more easily disrupt the posterior and inferior cerebellum due to this vessel’s later maturation [18, 23, 25, 26]. Our study suggests a need for a more careful examination of the peripheral and inferior parts of the cerebellum to improve the detection of type 3 cerebellar hemorrhage.

Some limitations of this study should be acknowledged such as the small sample size and the retrospective design. Furthermore, on retrospective review of US images, it was not clear whether the small hemorrhages were truly not visible on US or were simply not captured in the image. Additionally, the time interval between the US and MRI may have led to imaging in different stages of hemorrhage, including instances of new development or disappearance. The inherent heterogeneity in US images, due to the use of different machines and operators throughout the study period, along with unavoidable factors such as portable examination conditions, operator skills, and patient-specific acoustic windows, may have also affected the accuracy of lesion detection.

In this study, we introduced a new classification of cerebellar hemorrhage based on the MRI findings. The detection rates of cerebellar hemorrhages on US vary consistently across the types of cerebellar hemorrhage. Small type 1 cerebellar hemorrhages were generally not discernible on US. To avoid missing cerebellar hemorrhage on US, preterm infants, low birth weight infants, or infants with supratentorial abnormalities should be more carefully evaluated and the peripheral and inferior parts of the cerebellum should be scrutinized in order not to miss the type 3 cerebellar hemorrhage.

## Data Availability

The datasets generated during and/or analyzed during the current study are available from the corresponding author on reasonable request.
